# Clinico-radiological correlation with outcome in acute epidural haematoma: a tertiary centre experience from Nepal

**DOI:** 10.1097/MS9.0000000000002018

**Published:** 2024-04-04

**Authors:** Rupesh Chakradhar, Kayleigh Anjali Harrylal, Khusbu Kumari, Susmin Karki, Gopal Sedain, Amit Pradhanang, Sushil K. Shilpakar, Mohan Raj Sharma

**Affiliations:** a Department of Neurosurgery, Tribhuvan University Teaching Hospital; b University of Birmingham, Birmingham, UK; c Maharajgunj Medical Campus, Institute of Medicine, Kathmandu, Nepal

**Keywords:** epidural haematoma, modified Rankin scale, neurosurgery, traumatic brain injury

## Abstract

**Background:**

Epidural haematoma (EDH) accounts for up to 15% of severe traumatic brain injury (TBI) cases and remains the most common cause of mortality and disability. Several clinical and radiological factors affect patient outcomes. This study aims to correlate patients’ clinical and radiological profiles with acute EDH outcomes.

**Methods:**

A retrospective, single-centred, consecutive case series was conducted on the patients diagnosed with an acute EDH admitted to Tribhuvan University Teaching Hospital (TUTH) between May 2019 and April 2023. The modified Rankin scale (mRS) was used to assess the outcome. Univariate analysis and Kruskal–Wallis H test with Dunn-Bonferroni post-hoc test was conducted.

**Results:**

There were 107 patients diagnosed with EDH, of which 52.3% were less than 20 years old with male preponderance. Falls were the most common mechanism of injury (64.5%), and most cases were referred to, not brought directly. The majority had a GCS score greater than or equal to 13 (85%) at presentation, and only 5.5% had a GCS score less than or equal to 8. According to the mRS, most patients had favourable outcomes, with 88.7% having no significant disability and 11.3% having a slight disability.

**Conclusion:**

This case series is the largest and most recent report from Nepal and demonstrated that GCS, pupillary response, skull fracture, neurological symptoms, pre-hospital and intra-hospital delay, and management modalities are critical factors in determining the total hospital and ICU stay but did not have an impact on the mRS scores.

## Introduction

Highlights

- This is the largest case series available in the past two decades.
- The results reflect the presentation, management and outcome among EDH cases in low-income and middle-income countries.
- This paper serves as a foundation for future research and the development of targeted interventions to improve outcomes for EDH patients in low-income settings.

Traumatic brain injury (TBI) is a major cause of morbidity and mortality worldwide, as the annual incidence is estimated up to 69 million as of 2022^1,2^. TBI causes a serious socioeconomic problem due to the need for further care and rehabilitation^3^. Some major causes of TBI include falls, road traffic accidents (RTA), and physical assaults, with RTA being the greatest in Southeast Asia alongside Africa (both 56%)^4,5^. Up to 15% of severe TBI cases present as an epidural haematoma (EDH), which is an accumulation of blood in a potential space between the inner table of cranial bone and periosteal dura mater^6–9^. The Brain Trauma Foundation (BTF) recommends that patients with an acute EDH and a Glasgow Coma Scale (GCS) score less than 9 with anisocoria should undergo surgical evacuation as early as possible^10^. Craniotomy followed by haematoma evacuation is the mainstay treatment for acute and symptomatic EDHs^1^.

Early detection and timely surgical excision of radiographically substantial EDH are required to prevent a potentially fatal stage. Even so, EDH is still oddly portrayed as a neurosurgical emergency despite being recognised globally. There are limited studies from Southeast Asia correlating the clinico-radiological profiles and management strategies to the outcome of the patient with EDH. Hence, this case series aims to describe and correlate different clinic-radiological factors with the outcomes of patients with EDH.

## Materials and methods

After approval and ethical clearance from the Institutional Review Committee (IRC) of the Institute of Medicine (IOM), data was collected. This study is a retrospective, single-centred, and consecutive case series. Patients diagnosed with EDH and having a follow-up period of at least three months were identified from the neurosurgical database of inpatients who were admitted from May 2019 to April 2023 at our centre. The necessary data were extracted from the hospital records. The physical files obtained contained information on demographics, clinical factors, computed tomography (CT) scan features, management, and outcomes. Data were also collected from telephone calls with patients after a 3-month minimum follow-up period to determine the outcome using the modified Rankin scale (mRS)^11^.

The statistical analyses were conducted using the IBM Statistical Package for the Social Sciences (SPSS) Statistics, version 29.0.0.0 (241). The categorical variables were conveyed as counts and percentages, whereas the continuous variables were reported as medians.

A nonparametric test using the Kruskal–Wallis H test was conducted to determine if there were any statistically significant differences between independent variables and dependent variables. For the statistically significant results, the Dunn-Bonferroni post hoc test was performed.

For this case series, the Preferred Reporting of Case Series in Surgery (PROCESS) guideline was followed^12^.

## Results

### Demographic features

One hundred and seven (107) patients with EDH presented between May 2019 and April 2023. Amongst the patients, 78.5% were male and 21.5% were female. 52.3% of patients were less than 20 years old, and falls had the highest frequency regarding mechanism of injury (64.5%). More patients were referred from other hospitals (53.1%) than those who were brought directly (46.9%). Demographic details are shown in Table 1.

**Table 1:** Demographic features of patients presenting with EDH.

	Variable	Attribute	Frequency (Percent), *n* (%)	
Demographics	Sex	Male	84 (78.5)	
		Female	23 (21.5)	
		Total	107 (100)	
	Age (years)	<20	56 (52.3)	
		21–30	19 (17.8)	
		31–40	12 (11.2)	
		41–50	10 (9.3)	
		>50	10 (9.3)	
		Total	107 (100)	
	Mechanism of injury	RTA	21 (19.6)	
		Fall	69 (64.5)	
		Physical assault	10 (9.3)	
		Other	7 (6.5)	
		Total	107 (100)	
	Directly brought	Yes	45 (46.9)	
		No	51 (53.1)	
		Total	96 (100)	
		Median (IQR)	Minimum	Maximum
	Time from injury to hospital (h)	18 (16.5)	0.5	168
	Time from hospital to operating theatre (h)	6 (5.5)	1.5	144

EDH, epidural haematoma; IQR, interquartile range; RTA, road traffic accident.

The days in the hospital were statistically significant between those who arrived directly and those who were referred (*P*=0.008). The days in ICU and mRS scores were not statistically significantly different between the two groups.

The days in the hospital, ICU days, and mRS scores were statistically and significantly different between the different ages. But post hoc analysis revealed statistically significant differences between less than 20 (40.40) and 21–30 (63.31) (*P*=0.049), and less than 20 and 31–40 (77.29) (*P*=0.001), but not the other age combinations. Similarly, for days in ICU, the post hoc analysis revealed statistically significant differences between less than 20 (46.90) and 31–40 (71.42) (*P*=0.031), but not the other age combinations. However, in the mRS scores, no statistically significant differences were revealed from the post hoc analysis.

### Clinical profile

#### Physiological features

Most patients with EDH presented with a GCS score greater than or equal to 13 (85%). Only 5.5% (6/107) of patients had less than or equal to 8 GCS score. 44.9% of patients presented with loss of consciousness, and 9.3% presented with seizures. 15/106 (14.2%) displayed neurological symptoms such as abnormal pupillary response, altered sensation, abnormal speech, dysdiadochokinesia, upgoing plantar and muscle weakness. 69/107 (64.5%) patients with EDH presented with other symptoms such as vomiting, nausea, fractures, swelling, raccoon eye, headaches, dizziness, nasal bleeding, lacerations, and disc oedema with subconjunctival haemorrhage.

#### Laboratory parameters

Fifty percent (36/72) of patients had a prolonged PT/INR at presentation. Approximately 44% (36/81) of patients had leucocytosis, whereas 4.9% (4/81) presented with leukopenia. Twenty-five percent (12/48) patients were hyperglycaemic, and 2.1% (1/48) were hypoglycaemic (Table 2).

**Table 2:** Clinical features of patients presenting with EDH

	Variable	Attribute	Frequency (Percent), *n* (%)
Physiological features	Initial GCS	Mild (13–15)	91 (85)
		Moderate (9–12)	10 (9.3)
		Severe (3–8)	6 (5.5)
		Total	107 (100)
	Loss of consciousness	Yes	48 (44.9)
		No	59 (55.1)
		Total	107 (100)
	Seizure	Yes	10 (9.3)
		No	97 (90.7)
		Total	107 (100)
	Pupils	Abnormal	7 (6.7)
		Normal	98 (93.3)
		Total	105 (100)
	Neurological symptoms	Yes	15 (14.2)
		No	91 (85.8)
		Total	106 (100)
	Other symptoms	Yes	69 (64.5)
		No	38 (35.5)
		Total	107 (100)
Laboratory features	PT/INR	Normal	36 (50)
		Prolonged	36 (50)
		Total	72 (100)
	Total leucocyte count	Normal	41 (50.6)
		Leucocytosis	36 (44.4)
		Leukopenia	4 (4.9)
		Total	81 (100)
	Glucose	Normal	35 (72.9)
		Hyperglycaemia	12 (25)
		Hypoglycaemia	1 (2.1)
		Total	48 (100)

EDH, epidural haematoma; GCS, Glasgow Coma Scale; PT/INR, Prothrombin time/International normalized ratio.

The days in the hospital and ICU were statistically significant between the different initial GCS (*P*=0.001 and *P*<0.001, respectively) scores, abnormal pupillary responses (*P*=0.018 and *P*<0.001, respectively), and those who presented with neurological symptoms, and those who did not (*P*=0.002 and *P*=0.023, respectively), whereas the mRS scores were not statistically significant among the variables mentioned above.

The days in the hospital were statistically significant between those who presented with loss of consciousness and those who did not (*P*=0. 014). The days in ICU and mRS scores were not statistically significantly different between the two groups.

There were no statistically significant differences in outcome for the mechanism of injury, seizures, systemic symptoms, prothrombin time, total leucocyte count, blood glucose, location of EDH, presence of subdural haemmorhage (SDH), midline shift, contusion, and thickness of haematoma.

### Radiological profile

The frontal lobe was the most common location for EDH in patients in this study (21.6%). Amongst the patients, 12.1% (13/107) presented with subdural haemorrhage (SDH) alongside the EDH. The volume of EDH observed ranged from 2.4 mL to 100 ml. Around 30% (33/107) of CT scans noted the presence of skull fractures, and 13.1% (14/107) scans depicted contusion. Only 3.7% (4/107) of patients displayed a midline shift in their CT scans (Table 3).

**Table 3:** Radiological features of patients presenting with EDH

	Variable	Attribute	Frequency (Percent), *n* (%)	
CT scan characteristics	Location of EDH	Frontal	22 (21.6)	
		Parietal	20 (19.6)	
		Temporal	20 (19.6)	
		Occipital	11 (10.8)	
		Frontoparietal	11 (10.8)	
		Temporoparietal	3 (2.9)	
		Frontotemporal	7 (6.9)	
		Parieto-occipital	8 (7.8)	
		Total	102 (100)	
	Presence of SDH	Yes	13 (12.1)	
		No	94 (87.9)	
		Total	107 (100)	
	Skull fracture	Yes	33 (30.8)	
		No	74 (69.2)	
		Total	107 (100)	
	Midline shift	Yes	4 (3.7)	
		No	103 (96.3)	
		Total	107 (100)	
	Contusion	Yes	14 (13.1)	
		No	93 (86.9)	
		Total	107 (100)	
		Median (IQR)	Minimum	Maximum
	Thickness of haematoma (ml)	40 (30)	2.4	100

CT, computed tomography; EDH, epidural haematoma; IQR, interquartile range; SDH, subdural haemmorhage.

**Figure 1:**
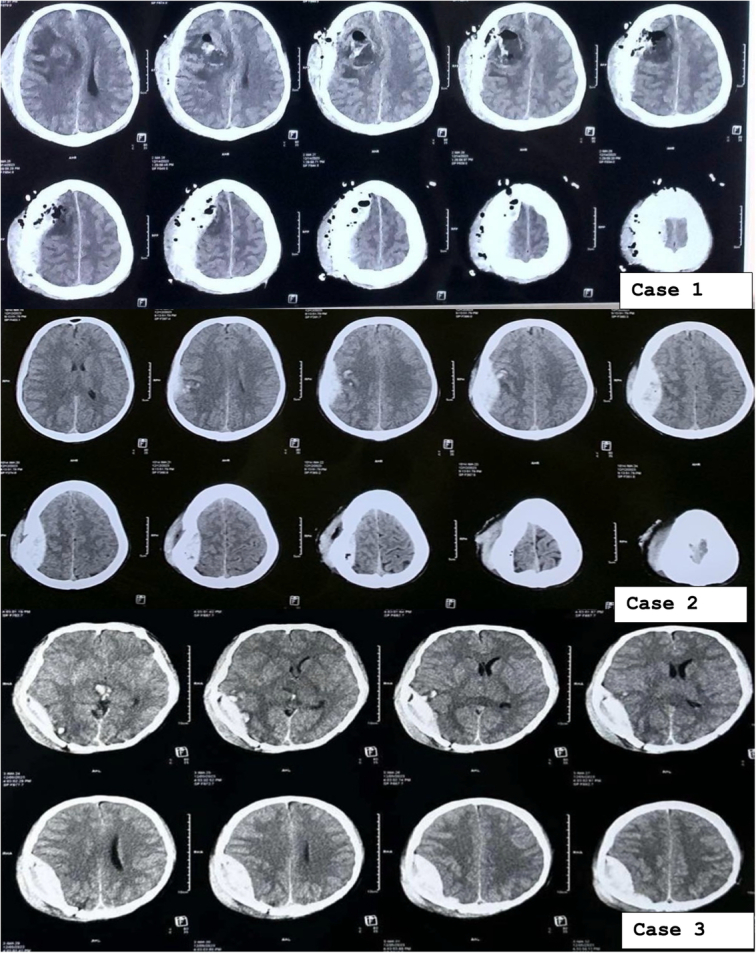
Computed tomography scans from three cases, each illustrating an epidural haematoma.

**Figure 2:**
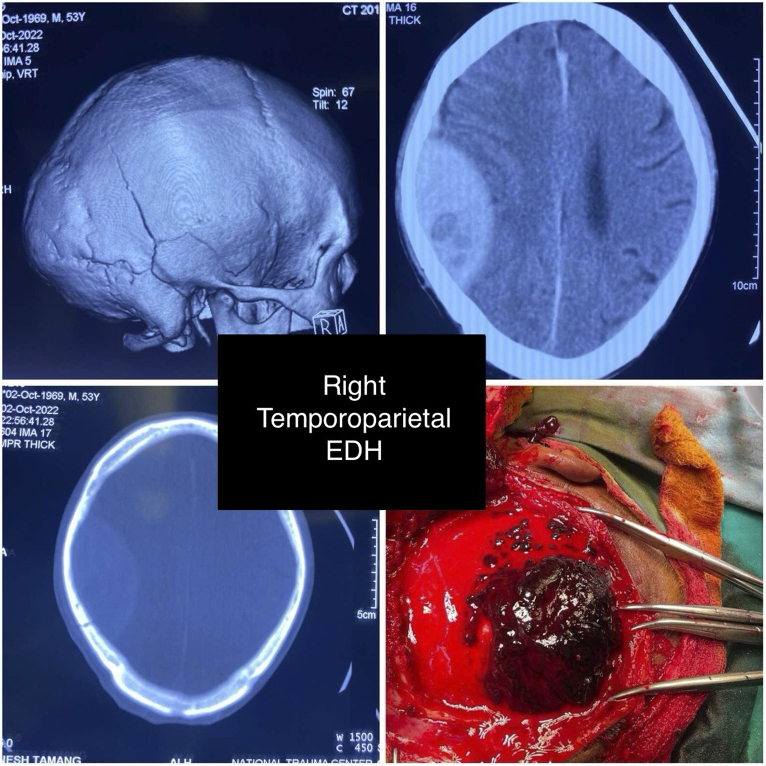
The image showcases a right temporoparietal epidural haematoma as revealed in both a computed tomography scan and during intraoperative exploration. EDH, epidural haematoma.

The days in the hospital were statistically significantly different between those who presented with skull fractures and those who did not (*P*=0. 044), whereas the days in ICU and mRS scores were not statistically significantly different between the two groups. See legends for the CT scan showing EDH (Figs. 1 and 2).

### Management and outcome

Fifty-seven percent (57/100) of patients underwent surgery, whereas 43% (43/100) of patients were managed conservatively. The median number of days spent in the hospital was six days [interquartile range (IQR)= 6], and the median number of days spent in the intensive care unit (ICU) was two days (IQR=3). The maximum number of days spent in the hospital was 62 days, and the maximum number of days spent in the ICU was 31 days.

Approximately 88% (94/106) scored one on the mRS scale after a minimum 3-month follow-up period. This score represents no significant disability. On the other hand, 11.3% (12/106) scored 2, which represents slight disability (Table 4).

**Table 4:** Hospital management features and outcome of patients with EDH

	Variable	Attribute	Frequency (Percent), n (%)	
Hospital Management	Management	Operative	57 (57)	
		Non-operative	43 (43)	
		Total	100 (100)	
		Median (IQR)	Minimum	Maximum
	Hospital stays	6 (6)	1	62
	ICU days	2 (3)	0	31
Outcome	mRS	1	94	88.7
		2	12	11.3
		Total	106	100

EDH, epidural haematoma; IQR, interquartile range; mRS, modified Rankin scale.

The days in the hospital and ICU were statistically significantly different between those who were managed operatively and those who were managed conservatively (*P*<0.001) whereas the mRS scores were not statistically significantly different between the two groups.

## Discussion

According to the available information, only a few studies compare clinico-radiological features and outcomes of patients with EDH from Southeast Asia and none from Nepal. Our study details the characteristics of EDH presentation and management and the impact of these features on outcomes.

### Demographic features

There was a higher incidence of EDH in males than females, which is consistent with other studies^13–15^. The male preponderance may be attributed to the early riding of unsupervised two-wheelers and more physical and outdoor sports activities than girls^16^. Younger patients (<20, 21–30 years) were mostly affected because the dura mater becomes more adherent to the cranium as we age, decreasing the risk of developing a haematoma in the epidural space^13^. This patient population also had better outcomes with fewer days in hospital and ICU, as well as a score of 1 on the mRS scale.

Falls were the most common mechanism of injury (64.5%), followed by road traffic accidents (RTA) (19.6%) and physical assaults (9.3%). This was similarly observed in a study conducted in Nepal and India^15,17–21^. In contrast, Umerani *et al*.^22^ found RTA to be the major mechanism of injury for EDH.

There is difficult terrain in Nepal, making transportation unavailable, difficult, and long. Hence, many people suffer from late hospital presentations, causing delays in definitive management. In developing countries, there is usually a delay in pre-hospital and intra-hospital care before patients are referred to the neurosurgical department^9,23^. A study conducted at a tertiary care centre noted a pre-hospital delay of up to 58 hours^23^. Patients’ visiting multiple hospitals was demonstrated to be an important reason for the delay in deliverance of surgical intervention^23^. There was a correlation between the number of days spent in the hospital with those who arrived directly and with those who were referred. More patients who were referred spent more days in the hospital.

### Clinical profile

The clinical features of the EDH include lucid interval, headache, vomiting, drowsiness, confusion, aphasia, pupillary abnormalities, focal deficits, such as hemiparesis, decerebration, and seizures or signs of increased intracranial pressures such as ipsilateral dilated pupil due to uncal herniation and Cushing reflex, that is hypertension, bradycardia, and irregular respiration, etc^7^.

Similar to our study, the study by Jamieson and colleagues and Sobti and colleagues also showed loss of consciousness as a most common presentation^15,24^. 44.9% of patients presented with loss of consciousness, and there was a statistically significant difference when correlated with the number of days in the hospital. None of our patients had lucid intervals. A systematic review and meta-analysis revealed that the mortality rate in patients with fixed pupils was 29.7% (95% CI 14.7–47.2%), and a favourable outcome was seen in 54.3% (95% CI 36.3–71.8%)^25^.

Despite having few abnormal pupillary responses (7/105), we had no mortality but rather spent more days in the hospital and ICU.

Aromatario and colleagues and Sobti and colleagues have reported that lower GCS at presentation correlates with an unfavourable outcome^15,26^. In contrast, in our case, only six patients had severe GCS scores and spent more days in the hospital than those with mild scores. Furthermore, the mRS scores after 3-month follow-up showed that most patients had favourable outcomes with no mortality.

### Radiological profile

CT of the head is commonly used to identify acute EDH with high diagnostic accuracy, showing classic brain CT scan features of biconvex or lens-shaped mass due to the limited ability of blood to expand within the fixed attachment of the dura to the cranial sutures which does not let to cross suture lines^27^. The temporal region is the classical location of EDH in adults, most commonly due to a tear of the middle meningeal artery and sometimes due to the middle meningeal vein. However, the source of bleeding differs according to the brain region involved^6,7^.

The frontal lobe was the most common anatomical location for EDH in our case. We differ from the studies by Sobti and colleagues, Gerlach and colleagues, Ben and colleagues, and Umerani and colleagues, who observed the parietal region as the commonest location of haematoma^15,22,28,29^. A study by Kandregula *et al*.^30^ analysed EDH among 201 children where the temporoparietal followed by the frontal region was the most common location for EDH. Even though there is a postulation that EDH in the temporal region might have increased mortality due to the risk of uncal herniation, none of our cases had such complications^31^. Also, the location of the bleed was not associated with the outcome.

In a series of 203 patients undergoing decompressive craniectomy for acute traumatic brain injury, the incidence of a delayed epidural haematoma complication was 6%, of which all had had a contralateral calvarial fracture^32^. In contrast, there was no delayed haematoma formation among our patients with skull fractures. There was a significant difference in the number of days spent in the hospital between those who presented with skull fractures and those who did not, where those with a skull fracture spent fewer days in the hospital.

There were no significant findings for correlations between CT scan characteristics of SDH, midline shift, contusion, and haematoma thickness (2.4–100 ml) with outcomes in this case series. However, haematoma volume greater than 30–150 ml and midline shift greater than 10 mm or 12 mm are known to correlate with poor prognosis^14^.

### Management

Surgical intervention is recommended in patients with acute EDH, haematoma volume greater than 30 ml regardless of GCS, and GCS less than 9 with pupillary abnormalities like anisocoria^7^. In our case, 57% of patients were managed surgically, and 43% of patients were managed conservatively. Patients who were managed surgically spent more days in the hospital and ICU than those who were managed conservatively without any statistical significance between the type of management when compared with mRS scores. In this study, patients had mostly favourable outcomes, with 88.7% scoring 1 (no significant disability) on the mRS scale and 11.3% scoring 2 (slight disability). Though there were no correlations between mRS score and type of management in this study, other studies have found that patients who underwent surgery also had good outcomes for EDH^33,34^.

### Limitations

The patient’s cognitive functions could not be assessed, and the minimum follow-up period was only 3 months. The Kruskal–Wallis test is insensitive to outliers and is limited to comparing more than two groups.

## Conclusion

This case series is the largest and most recent report from Nepal. It demonstrated that younger ages, skull fractures, and conservative management were associated with better outcomes, whereas older ages, lower GCS score, loss of consciousness, abnormal pupillary response, neurological symptoms, and surgical management were associated with unfavourable outcomes in terms of the hospital or ICU stay but did not affect the mRS score at 3 months. This paper is a foundation for future research and developing targeted interventions to improve outcomes for EDH patients in low-income settings.

## Ethical approval

We had obtained ethical clearance from the institutional review committee (IRC), Institute of Medicine, prior to the collection of the data.

## Consent

As, this is a retrospective case series, which did not require the direct patient involvement directly so patient consent is not needed. And, the patient’s identity has been kept hidden.

## Source of funding

None.

## Author contribution

R.C.: drafting original manuscript, revision of manuscript. K.A.H. data collection, data analysis, drafting original manuscript, revision of manuscript. K.K. and S.K.: data collection, drafting original manuscript, revision of manuscript. M.R.S.: conceptualisation and revision of manuscript. S.S., A.P., and G.S.: revision of manuscript.

## Conflicts of interest disclosure

The author(s) declare(s) that there is no conflict of interest regarding the publication of this paper.

## Research registration unique identifying number (UIN)

1. Name of the registry: None.
2. Unique Identifying number or registration ID: None.
3. Hyperlink to your specific registration (must be publicly accessible and will be checked): None.

## Guarantor

Rupesh Chakradhar.

## Data availability statement

All the data generated during this study can be accessed through direct communication with the corresponding author and the agreement of all research team members.

## Provenance and peer review

Not commissioned, externally peer-reviewed.

## References

[1] James SL Bannick MS Montjoy-Venning WC. Global, regional, and national burden of traumatic brain injury and spinal cord injury, 1990-2016: a systematic analysis for the Global Burden of Disease Study 2016. Lancet Neurol 2019;18:56–87. 30497965 10.1016/S1474-4422(18)30415-0 PMC6291456

[2] Maas AIR Menon DK David Adelson PD. Traumatic brain injury: integrated approaches to improve prevention, clinical care, and research. Lancet Neurol 2017;16:987–1048. 29122524 10.1016/S1474-4422(17)30371-X

[3] Koskinen S Alaranta H. Traumatic brain injury in Finland 1991-2005: a nationwide register study of hospitalized and fatal TBI. Brain Inj 2008;22:205–214. 18297592 10.1080/02699050801938975

[4] Lafta G Sbahi H. Factors associated with the severity of traumatic brain injury. Med Pharm Rep 2023;96:58–64. 36818327 10.15386/mpr-2314 PMC9924815

[5] Dewan MC Rattani A Gupta S. Estimating the global incidence of traumatic brain injury. J Neurosurg 2019;130:1080–1097. 10.3171/2017.10.JNS17352 29701556

[6] Langlois JA Rutland-Brown W Wald MM. The epidemiology and impact of traumatic brain injury: a brief overview. J Head Trauma Rehabil 2006;21:375–378. 16983222 10.1097/00001199-200609000-00001

[7] Bullock MR Chesnut R Ghajar J. Surgical management of traumatic parenchymal lesions. Neurosurgery 2006;58(suppl 3):S2-25-S2-46. doi:10.1227/01.NEU.0000210365.36914.E3 16540746

[8] Mishra A Mohanty S. Contre-coup extradural haematoma: a short report. Neurol India 2001;49:94–95. 11303253

[9] Mezue W Ndubuisi C Chikani M. Traumatic extradural hematoma in Enugu, Nigeria. Nigerian J Surg 2012;18:80. 10.4103/1117-6806.103111 PMC3762009 24027399

[10] Bullock MR Chesnut R Ghajar J. Surgical management of acute subdural hematomas. Neurosurgery 2006;58(suppl_3):S2-16–S2-24. 16710968

[11] Quinn TJ Dawson J Walters MR. Reliability of the modified Rankin Scale. Stroke 2009;40:3393–3395. 19679846 10.1161/STROKEAHA.109.557256

[12] Agha RA Sohrabi C Mathew G. The PROCESS 2020 Guideline: Updating Consensus Preferred Reporting Of CasE Series in Surgery (PROCESS) Guidelines. Int J Surg 2020;84:231–235. 33189880 10.1016/j.ijsu.2020.11.005

[13] Chicote Álvarez E González Castro A Ortiz Lasa M. Epidemiology of traumatic brain injury in the elderly over a 25 year period. Revista Española de Anestesiología y Reanimación (English Edition) 2018;65:546–551. 30054092 10.1016/j.redar.2018.06.003

[14] Khairat A Waseem M. Epidural Hematoma. In: StatPearls [Internet]. Treasure Island (FL): StatPearls Publishing; 2024. Accessed July 31, 2023.. https://www.ncbi.nlm.nih.gov/books/NBK518982/30085524

[15] Sobti S Goyari M Harpanahalli R. Clinico-radiological correlation with outcome in traumatic pediatric extradural hematoma: a single institutional experience. J Pediatr Neurosci 2021;16:113. 35018178 10.4103/jpn.JPN_61_20 PMC8706599

[16] Zhong W Sima X Huang S. Traumatic extradural hematoma in childhood. Child’s Nerv Syst, 29:635–641. 23239253 10.1007/s00381-012-1971-x

[17] Dahal S Shrestha DK Pradhanang AB. Demography and outcome of pediatric traumatic brain injury; experience from a university teaching hospital in Nepal. J Inst Med Nepal 1 Inst Med, Kathmandu, Nepal 2022;44:53–58.

[18] Mukhida K Sharma MR Shilpakar SK. Pediatric neurotrauma in Kathmandu, Nepal: implications for injury management and control. Child’s Nerv Syst 2006;22:352–362. 16170573 10.1007/s00381-005-1235-0

[19] Agrawal A Agrawal C Kumar A. Epidemiology and management of paediatric head injury in eastern Nepal. African J Paediatr Surg 2008;5:15. doi:10.4103/0189-6725.41630 19858657

[20] Karmacharya BG Acharya B. Pediatric head injuries in a neurosurgery center of Nepal: an epidemiological perspective. Am J Public Health Res 2015;3:76–79.

[21] Garg K Sharma R Gupta D. Outcome predictors in pediatric head trauma: a study of clinicoradiological factors. J Pediatr Neurosci 2017;12:149. 28904572 10.4103/jpn.JPN_179_16 PMC5588639

[22] Umerani MS Abbas A Aziz F. Pediatric extradural hematoma: clinical assessment using King’s outcome scale for childhood head injury. Asian J Neurosurg 2018;13:681. 30283526 10.4103/ajns.AJNS_164_16 PMC6159040

[23] Sah H Shrestha D Rajbhandari B. Temporal delay in neurosurgical patients and outcome in a tertiary care center in Nepal. J Nepal Health Res Counc 2021;19:170–174. 33934154 10.33314/jnhrc.v19i1.3429

[24] Jamieson KG Yelland JD. Extradural hematoma. Report of 167 cases. J Neurosurg 1968;29:13–23. 5302643 10.3171/jns.1968.29.1.0013

[25] Scotter J Hendrickson S Marcus HJ. Prognosis of patients with bilateral fixed dilated pupils secondary to traumatic extradural or subdural haematoma who undergo surgery: a systematic review and meta-analysis. Emerg Med J 2015;32:654–659. 25385844 10.1136/emermed-2014-204260

[26] Aromatario M Torsello A D’Errico S. Traumatic epidural and subdural hematoma: epidemiology, outcome, and dating. Medicina (B Aires) 2021;57:125. 10.3390/medicina57020125 PMC7912597 33535407

[27] Shetty SP Chandrappa A Das SK. Evaluation of sequential head computed tomography in traumatic brain injuries. Cureus 2022;14:e27772. 36106236 10.7759/cureus.27772 PMC9449334

[28] Ben Abraham R Lahat E Sheinman G. Metabolic and clinical markers of prognosis in the era of CT imaging in children with acute epidural hematomas. Pediatr Neurosurg 2000;33:70–75. 11070432 10.1159/000028990

[29] Gerlach R Dittrich S Schneider W. Traumatic epidural hematomas in children and adolescents: outcome analysis in 39 consecutive unselected cases. Pediatr Emerg Care 2009;25:164–169. 19262419 10.1097/PEC.0b013e31819a8966

[30] Kandregula S Sadashiva N Konar S. Surgical management of traumatic extradural hematomas in children: an analysis of 201 patients at a tertiary neurosurgical center. Childs Nerv Syst 2019;35:807–813. 30796557 10.1007/s00381-019-04088-1

[31] Rivas JJ Lobato RD Sarabia R. Extradural hematoma: analysis of factors influencing the courses of 161 patients. Neurosurgery 1988;23:44–51. 3173664 10.1227/00006123-198807000-00010

[32] Talbott JF Gean A Yuh EL. Calvarial fracture patterns on CT imaging predict risk of a delayed epidural hematoma following decompressive craniectomy for traumatic brain injury. AJNR Am J Neuroradiol 2014;35:1930–1935. 24948502 10.3174/ajnr.A4001 PMC7966253

[33] Tierney KJ Nayak N V Prestigiacomo CJ. Neurosurgical intervention in patients with mild traumatic brain injury and its effect on neurological outcomes. J Neurosurg 2016;124:538–545. 26406795 10.3171/2015.4.JNS142440

[34] Mejaddam AY Elmer J Sideris AC. Prolonged emergency department length of stay is not associated with worse outcomes in traumatic brain injury. J Emerg Med 2013;45:384–391. 23769388 10.1016/j.jemermed.2013.04.015

